# DNA methyltransferase inhibitors in hematological malignancies and solid tumors

**DOI:** 10.1002/ijc.70197

**Published:** 2025-11-07

**Authors:** Valentin Wenger, Guillermo Garcia‐Manero, Robert Zeiser, Michael Lübbert

**Affiliations:** ^1^ Department of Medicine I (Division of Hematology, Oncology and Stem Cell Transplantation) University of Freiburg Medical Center, Faculty of Medicine Freiburg Germany; ^2^ Department of Leukemia The University of Texas MD Anderson Cancer Center Houston Texas USA; ^3^ German Cancer Consortium (DKTK), Partner Site Freiburg, a Partnership Between DKFZ and University of Freiburg Medical Center Freiburg Germany; ^4^ Signaling Research Centres BIOSS and CIBSS – Centre for Integrative Biological Signaling Studies, University of Freiburg Freiburg Germany; ^5^ German Society of Hematology and Medical Oncology (DGHO) Working Group ‘Clinical and Translational Epigenetics’ Germany

**Keywords:** differentiation, epigenetic therapy, hypomethylating agents, retinoic acid, venetoclax

## Abstract

Epigenetic modifications such as DNA methylation play a fundamental role in oncogenesis and the progression of neoplasms neoplasias. DNA methyltransferase inhibitors (DNMTi) constitute a family of therapeutic agents that impede the methylation at the 5‐position on cytosine nucleotides, thereby modulating the epigenetic regulation of tumor suppressor genes, oncogenes, and other key regulatory genes. The first‐generation DNMTi azacitidine and decitabine have demonstrated substantial efficacy in the treatment of medically non‐fit, older patients with acute myeloid leukemia (AML) or myelodysplastic syndrome (MDS) ineligible for intensive chemotherapy (IC), by virtue of their favorable safety profile. Despite these clinical achievements, however, single‐agent DNMTi treatment has faced challenges such as limited, non‐durable response rates and remissions as well as the emergence of secondary resistance. These limitations have driven broad efforts to identify more effective, dual treatment combinations, such as now attained with the DNMTi‐BCL‐2 (B‐cell lymphoma 2) inhibitor combination. This review aims to provide a comprehensive overview and analysis of the pivotal role of DNMTi in both mono‐ and combination therapies for myeloid malignancies over the last 40 years, while also exploring their potential applicability in lymphoid malignancies. Additionally, this review assesses the therapeutic potential of DNMTi in the management of solid tumors. Through these discussions, we intend to enhance the understanding of the mechanistic and therapeutic implications of DNMTi across a diverse array of malignancies.

## Graphical abstract

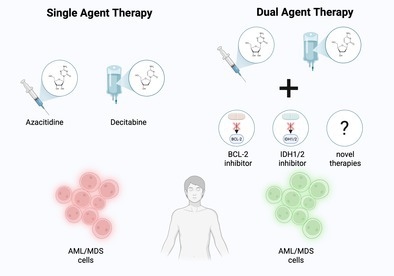

**Abbreviations**
7 + 3: cytarabine and anthracycline (daunorubicin/idarubicin/mitoxantrone) based standard induction therapy
AE: adverse events
Allo‐HCT: allogeneic hematopoietic stem cell transplantation
AML: acute myeloid leukemia
ATRA: all‐trans retinoic acid
BCL‐2: B‐cell lymphoma 2
BSC: best supportive care
BTK: Bruton's tyrosine kinase
CALGB: Cancer and Leukemia Group B
CAR: chimeric antigen receptor
CCR: conventional care regimen
CD: cluster of differentiation
CLL: chronic lymphocytic leukemia
CMML: chronic myelomonocytic leukemia
CR_(i)_
: complete remission_(incomplete)_
CRL: cullin‐RING ligases
CTLA‐4: cytotoxic T‐lymphocyte associated protein 4
DFS: disease‐free survival
DLI: donor lymphocyte infusion
DNMT(i): DNA methyltransferase (inhibitor)
EFS: event‐free survival
EMA: European Medicine Agency
EORTC: European Organisation for Research and Treatment of Caner
FDA: U.S. Food and Drug Administration
FLT3: Fms‐like tyrosine kinase 3
G‐CSF: granulocyte‐colony stimulating factor
GI: gastrointestinal
GIMEMA: Gruppo Italiano Malattie EMatologiche dell 'Adulto
GvHD: graft‐versus‐host disease
GvL: graft‐versus‐leukemia
HDACi: histone deacetylase inhibitor
IC: intensive chemotherapy
IDH1/2: isocitrate dehydrogenase 1/2
JMML: juvenile myelomonocytic leukemia
LSC: leukemia stem cell
MCL‐1: myeloid cell leukemia protein‐1
MDS: myelodysplastic syndrome
MPN: myeloproliferative neoplasm
MRD: measurable residual disease
NCI: National Cancer Institute
NHL: non‐hodgkin lymphoma
NPM1: nucleophosmin 1
NSCLC: non‐small cell lung cancer
ORR: overall response rate
OS: overall survival
PD‐(L)1: programmed cell death 1 (ligand 1) protein
PFS: progression‐free survival
PR: partial remission
QoL: quality of life
r/r: relapsed and refractory
RBC: red blood cell
RFS: relapse‐free survival
SAL: study alliance leukemia
SIRPα: signal regulatory protein α
(s)TIL: (stromal) tumor‐infiltrating lymphocytes
TC: treatment of choice
TET2: Tet‐methylcytosine‐dioxygenase 2
TI: transfusion independency
TIM‐3: T‐cell immunoglobulin domain and mucin domain‐3
TNBC: triple‐negative breast cancer
TP 53: tumor protein 53

## 1 INTRODUCTION

With the updated 2022 version of the “Hallmarks of Cancer”, epigenetics has been recognized as a fundamental cornerstone in the understanding of oncogenesis and tumor progression.^1^ This recognition is the result of more than 50 years of extensive research spanning from the initial discovery of gene regulatory processes and culminating in the development of novel targeted therapies that modulate these pathways.^2^, ^3^ Probably the most extensively studied epigenetic modifications is DNA methylation, a biochemical reaction in which a methyl residue is transferred to the 5 position of a cytosine nucleotide, yielding 5‐methylcytosine. While the majority of the genome in adult eukaryotic cells is methylated, methylation at CpG islands—often found within promoter regions—shows the strongest association to transcriptional regulation, thereby determining whether genes are activated or silenced.

The regulation of DNA methylation is orchestrated by a specific set of enzymes known as DNA methyltransferases (DNMT), which include DNMT1, DNMT3A, and DNMT3B. DNMT1 serves a dual role, maintaining methylation patterns during DNA replication, thereby ensuring the fidelity of epigenetic information through cellular divisions, but also serves as a transcriptional corepressor integrated into lineage master transcription factor hubs to regulate gene expression.^4^, ^5^, ^6^ By contrast, DNMT3A and DNMT3B establish “de novo” methylation patterns during development and in response to environmental stimuli. In cancer, dysregulation of DNA methylation is a frequent occurrence, with profound implications for cellular function. Aberrant methylation patterns can lead to the silencing of tumor suppressor genes, the activation of oncogenes, and the disruption of other regulatory genes essential for cell differentiation and genomic stability. A refined understanding of these mechanisms has paved the way for the development of therapies aimed at DNMT inhibition. Initially, this approach was primarily conceptualized as a way to reverse pathological hypermethylation and thereby reactivate silenced tumor suppressor genes; however, it has become increasingly clear that the clinical efficacy of DNMT inhibition does not appear to rely predominantly on such targeted gene reaction. Rather, current concepts suggest that cancer cells are highly dependent on the maintenance of a tightly controlled DNA methylation program and therefore particularly vulnerable to global perturbations in methylation.^7^, ^8^ The ultimate goal remains to exploit these vulnerabilities while minimizing the toxicities typically associated with standard, intensive chemotherapy (IC).

### 1.1 Azacitidine: preclinical development

Azacitidine (5‐azacytidine, 5‐azaCR) was first synthesized in Czechoslovakia about 60 years ago by František Šorm and his team when they were exploring a variety of antineoplastic agents.^9^, ^10^ Chemically, it differs from the naturally occurring pyrimidine nucleoside cytidine only by having the fifth carbon in cytidine replaced by nitrogen. It can be described as 4‐amino‐1‐b‐D‐ribofuranosyl 1–1,3,5 triazine‐2‐one or 1‐b‐D‐ribofuranosyl‐5‐azacytosine; C_8_H_12_N_4_O_5_ and has a molecular weight of 244 g/mol.^11^

Azacitidine, together with the structurally related agent decitabine, is a prodrug and belongs to the class of cytosine analogues.^12^ Following their cellular uptake by nucleoside specific transporters, azacitidine (and decitabine) requires sequential phosphorylation by key pyrimidine metabolism enzymes to become metabolically active.^13^ Azacitidine's main anticancer effects are assumed to be related to two distinct modes of action. After cellular intake and phosphorylation, azacitidine is incorporated mostly (80%–90%) into RNA, which leads to disruption of cellular and nuclear RNA metabolism and, consequently, disruption of protein synthesis.^14^, ^15^, ^16^ The effect of azacitidine on RNA function and protein synthesis is likely responsible for the liver and kidney toxicities observed at high doses in early clinical studies.^17^, ^18^ However, apart from RNA incorporation, a minor fraction of azacitidine (10%–20%) is also incorporated into DNA (via ribonucleotide reductase). In 1980, azacitidine's distinct mode of action, that is, its DNA demethylating activity, was first described.^19^ After DNA incorporation, azacitidine irreversibly binds to DNMT1, which results in intracellular depletion of the enzyme. Consequently, demethylation results in the reactivation and thus transcription of previously silenced genes, such as tumor suppressor genes.^20^, ^21^

By the late 1980s, it had become evident that aberrant DNA methylation constitutes a fundamental driver in the pathogenesis of most if not all neoplasias, including myeloid malignancies such as myelodysplastic syndrome (MDS) and acute myeloid leukemia (AML).^22^, ^23^, ^24^ The pronounced efficacy of DNMTi in hematological malignancies is largely attributed to the comparatively severalfold higher expression of pyrimidine metabolism in myeloid cells. In contrast, other cell types such as lymphoid cells display enhanced expression of enzymes involved in the de novo pyrimidine synthesis, resulting in increased intracellular pyrimidine pools that compete with DNMTi incorporation and thereby attenuate their therapeutic activity.^25^ At the clinically applied lower doses today, this demethylating effect is considered the main contributor to its activity rather than direct cytotoxicity.^26^, ^27^, ^28^

### 1.2 Azacitidine and decitabine: clinical development for the treatment of MDS and AML

#### 1.2.1 Azacitidine in MDS

It took almost another two decades from the landmark discovery of the DNA‐demethylating effects of DNMTi to the initiation of large clinical studies in MDS, with the first larger trials conducted by the Cancer and Leukemia Group B (CALGB).^29^ Their seminal clinical successes have paved the way for monotherapy approaches in MDS and AML (Table 1 summarizes single‐agent therapy randomized clinical trials; Table 3 summarizes all FDA/EMA approved single/combination therapies), replacing the former standard of “sole best supportive care” for patients who are not candidates for intensive therapies in AML or MDS.^30^, ^31^

**TABLE 1:** Clinical phase I–III trial overview of DNMTi single agent therapies in hematological malignancies.

Drugs and dosages/control arm	Study name and trial number	Entity	Phase	Number of patients	Results and outcomes	Quality of life	Citation
Azacitidine s.c. (75 mg/m^2^ for 7d q4w) vs. BSC	CALBG 9221	Several subtypes of MDS, (AML) and CMML	III	191	Improvement in ORR (60% vs. 5%), mOS (18 vs. 11 months) as well as reduced rates of transformation to AML (15% vs. 38%) in the azacitidine cohort compared to supportive care.	EORTC C30 and MHI—Treatment with azacitidine resulted in significantly improved fatigue, physical functioning dyspnea, psychosocial distress and positive affection compared to BSC.	LR Silverman et al. JCO 2002: Randomized controlled trial of azacitidine in patients with the myelodysplastic syndrome: a study of the cancer and leukemia group B
Azacitidine s.c. (75 mg/m^2^ for 7d q4w) vs. BSC	CALBG 9221 (subgroup analysis)	Several subtypes of MDS, (AML) and CMML	III	191	Patients receiving azacitidine displayed improved fatigue, physical functioning, dyspnea, positive affect and psychological distress compared to BSC.	EORTC C30 and MHI—Treatment with azacitidine resulted in significantly improved fatigue, physical functioning dyspnea, psychosocial distress and positive affection compared to BSC.	Kornblith AB et al. JCO 2002: Impact of Azacytidine on the Quality of Life of Patients with Myelodysplastic Syndrome Treated in a Randomized Phase III Trial: A Cancer and Leukemia Group B Study
Azacitidine s.c. (75 mg/m^2^ for 7d q4w) vs. CCR (BSC, low‐dose Ara‐C or IC)	AZA‐001 NCT00071799	Higher‐risk MDS	III	358	Improvement of mOS (24.5 vs. 15.0 months) and 2‐year survival rate (50.8% vs. 26.2%) with azacitidine compared to CCR.	N.a.	Fenaux P et al. Lancet Oncology 2009: Efficacy of azacitidine compared with that of conventional care regimens in the treatment of higher‐risk myelodysplastic syndromes: a randomized, open‐label, phase III study
Azacitidine s.c. (75 mg/m^2^ for 7d q4w) vs. CCR (BSC, low‐dose Ara‐C or IC)	AZA‐001 NCT00071799 (subgroup analysis)	Oligoblastic AML (20–30% blasts)	III	113	Improvement of mOS (24.5 vs. 16.0 months) and 2‐year survival rate (50% vs. 16%) of azacitidine compared to CCR.	N.a.	Fenaux P et al. JCO 2010: Azacitidine prolongs overall survival compared with conventional care regimens in elderly patients with low bone marrow blast count acute myeloid leukemia
Azacitidine s.c. (75 mg/m^2^ for 7d q4w) vs. CCR (BSC, low‐dose Ara‐C or IC)	AZA‐AML‐001 NCT01074047	AML (>30% blasts)	III	488	Augmented mOS (10.4 vs. 6.5 months) and 1‐year survival rate (46.5% vs. 34.2%) in patients receiving azacitidine compared to CCR.	EORTC C30 —Improvement of HRQoL in both arms without deterioration in primary or secondary QLQ C30 domains.	Dombret H et al. Blood 2015: International phase 3 study of azacitidine vs. conventional care regimens in older patients with newly diagnosed AML with>30% blasts
Azacitidine s.c. (50 mg/m^2^ for 5d q4w) vs. observation	HOVON97 NTR1810, 2008–001290‐15 (maintenance study)	Higher‐risk MDS and AML (>20% blasts) in CR after IC	III	116	Significant improvement of 12‐months DFS (64% vs. 42%) in high‐risk MDS/AML patients who achieved CR after 2 cycles of IC and subsequently received an azacitidine maintenance, compared to observation only.	N.a.	Huls G et al. Blood 2019: Azacitidine maintenance after intensive chemotherapy improves DFS in older AML patients
Azacitidine s.c. (75 mg/m^2^ for 7d q4w)	RELAZA NCT00422890 (relapse study)	Higher‐risk MDS and AML after allo‐HCT	II	20	Azacitidine treatment in MRD positive higher‐risk MDS/AML patients after allo‐HCT resulted in an ORR of 80% in donor chimerism in the absence of relapse and delayed subsequent relapse by a median of 231 days.	N.a.	Platzbecker U et al. Leukemia 2012: Azacitidine for treatment of imminent relapse in MDS or AML patients after allogeneic HSCT: results of the RELAZA trial
Azacitidine s.c. (75 mg/m^2^ for 7d q4w)	RELAZA2 NCT01462578 (relapse study)	MDS and AML after either IC or allo‐HCT with an established MRD marker	II	53	MRD positive advanced MDS/AML patients receiving azacitidine had a 12‐months RFS of 46% if remained MRD positive upon azacitidine treatment in contrast up to 88% if converted to MRD negativity.	N.a.	Platzbecker U et al. Lancet Oncology 2018: Measurable residual disease‐guided treatment with azacitidine to prevent hematological relapse in patients with myelodysplastic syndrome and acute myeloid leukaemia (RELAZA2): an open‐label, multicenter, phase 2 trial
Azacitidine s.c. (32 mg/m^2^ for 5d q4w) vs. observation	VZ‐AML‐PI‐0129 NCT00887068 (post allo‐HCT maintenance)	Higher‐risk MDS, AML and CMML after allo‐HCT	III	187	No statistically significant changes in median RFS (2.07 vs. 1.28 years) and OS (2.52 vs. 2.56 years) between azacitidine maintenance and observation only.	N.a.	Oran B et al. Blood advances 2020: A phase 3 randomized study of 5‐azacitidine maintenance vs. observation after transplant in high‐risk AML and MDS patients
Azacitidine s.c./i.v. (75 mg/m^2^ for 3d q4w) vs. decitabine i.v. (20 mg/m^2^ for 3d q4w)	NCT01720225	Lower‐risk MDS, MDS/MPN and CMML	II	113	ORR of 70% for decitabine arm compared to 49% for the azacitidine arm. No statistically significant improvement in transfusion independence (32% decitabine vs. 16% azacitidine) nor overall mEFS between decitabine and azacitidine (20 vs. 13 months).	N.a.	Jabbour E et al. Blood 2017: Randomized phase 2 study of low‐dose decitabine vs. low‐dose azacitidine in lower‐risk MDS and MDS/MPN
Decitabine s.c./i.v. (20 mg/m^2^ for 5d q4w (s.c.or i.v.) OR 10 mg/m^2^ for 10d q4w (i.v.))	N.a. (dose finding study)	Intermediate‐/Higher‐risk MDS and CMML	I/II	95	Higher dose i.v. decitabine was superior to higher dose s.c. decitabine or lower dose i.v. decitabine in inducing CR (39% vs. 21% vs. 24%) and hypomethylation.	N.a.	Kantarjian H et al. Blood 2007: Results of a randomized study of 3 schedules of low‐dose decitabine in higher‐risk myelodysplastic syndrome and chronic myelomonocytic leukemia
Decitabine i.v. (20 mg/m^2^ tid for 3d q6w) vs. BSC	N.a.	Several subtypes of MDS	III	170	Significantly higher ORR (17% vs. 0%) in MDS/MPN patients treated with decitabine compared to BSC.	EORTC C30—Treatment with decitabine resulted in improved global health status, fatigue and dyspnea.	Kantarjian H et al. Cancer 2006: Decitabine improves patient outcomes in myelodysplastic syndromes: results of a phase III randomized study
Decitabine i.v. (20 mg/m^2^ for 5d q4w) vs. TC (BSC or low‐dose Ara‐C)	DACO‐016	AML (≥ 20% blasts)	III	485	Significant increase in CR rates with decitabine treatment compared to TC (17.8% vs. 7.8%). No differences in mOS between the two available treatments.	N.a.	Kantarjian H et al. JCO 2012: Multicenter, randomized, open‐label, phase III trial of decitabine versus patient choice, with physician advice, of either supportive care or low‐dose cytarabine for the treatment of older patients with newly diagnosed acute myeloid leukemia
Decitabine i.v. (15 mg/m^2^ tid for 3d q6w) vs. BSC	EORTC‐06011 NCT00043134	Intermediate‐/Higher‐risk MDS and CMML	III	233	PFS was significantly improved with decitabine compared to BSC (6.6 vs. 3.0 months). No statistically significant changes in OS and AMLFS.	EORTC C30—Patients receiving decitabine displayed improvement in fatigue and physical functioning.	Lübbert M et al. JCO 2011: Low‐dose decitabine versus best supportive care in elderly patients with intermediate‐or high‐risk myelodysplastic syndrome (MDS) ineligible for intensive chemotherapy: final results of the randomized phase III study of the European Organisation for Research and Treatment of Cancer Leukemia Group and the German MDS Study Group
Decitabine i.v. (20 mg/m^2^ for 5d OR 10d q4‐8w for 3 cycles)	NCT01786343	AML	II	71	Comparable cCR response rates (43% vs. 40%) between the 5‐ and 10‐day treatment schedule as well as identical 1‐year OS (25% both). However, Huls et al. raised concerns about the equivalence due to an underpowered study design Huls, Gerwin, et al. “10‐day vs. 5‐day decitabine: equivalence cannot be concluded.” *The Lancet Haematology* 6.4 (2019): e177.	N.a.	Short NJ et al. Lancet Hematology 2019: Treatment with a 5‐day versus a 10‐day schedule of decitabine in older patients with newly diagnosed acute myeloid leukaemia: a randomized phase 2 trial
Decitabine (20 mg/m^2^ for 10d q4w (cycle 1) followed by 5/10d (cycle 2+)) vs. IC	EORTC‐130/AML21 NCT02172872	AML	III	606	While the 4‐year OS (26% vs. 30%) was similar between the decitabine and IC cohort, the decitabine arm displayed a better safety profile (infections, mucositis, diarrhea) with less adverse events grade ≥3.	EORTC C30 and ELD14—Patients who were receiving IC experience significant deterioration in physical functioning, role functioning, disease burden and fatigue after allo‐HCT. In contrast, the patients treated with decitabine did not report any significant reduction in HRQoL among any of these categories.	Lübbert M et al. Lancet Hematology 2023: 10‐day decitabine versus 3 + 7 chemotherapy followed by allografting in older patients with acute myeloid leukaemia: an open‐label, randomized, controlled, phase 3 trial Efficace F et al., Blood 2022:^143^ 10‐day decitabine versus intensive chemotherapy followed by transplantation in fit AML patients aged ≥60 years: health‐related quality of life outcomes of the randomized phase III trial AML21 of the EORTC Leukemia Group, Gimema, Celg, and Gmds‐SG.
Decitabine i.v. (20 mg/m^2^ for 10d q4w) vs. IC	EORTC‐130/AML21 NCT02172872 (subgroup analysis)	AML	III	130	MRD negativity rates in newly diagnosed AML patients receiving decitabine or IC were comparable after cycle 3/consolidation (14% vs. 19%). At 6 (18% vs. 27%), 12 (21% vs. 20%) and 18 (21% vs. 11%) months, patients who were either MRD negative or received allo‐HCT and were alive without progression were comparable.	N.a.	Venditti A et al. Blood 2023: Measurable Residual Disease Assessment in Patients with Acute Myeloid Leukemia Aged ≥60 Years Treated with a 10‐Day Decitabine Schedule Versus Intensive Chemotherapy in the AML21 Study (NCT02172872)
Decitabine i.v. (20 mg/m^2^ for 3d q4w for 1y) vs. observation	ECOG‐ACRIN (E‐A) E2906 study—part 3 (maintenance study)	AML	II	120	Part three of the ECOG‐ACRIN (E‐A) E2906 trial. Decitabine maintenance after IC in AML was associated with improved HR for OS and a trend toward higher DFS compared to observation only. Parent trial was terminated early.	N.a.	JM Foran et al. ASH 2019: Maintenance Decitabine (DAC) Improves Disease‐Free (DFS) and Overall Survival (OS) after Intensive Therapy for Acute Myeloid Leukemia (AML) in Older Adults, Particularly in FLT3‐ITD‐Negative Patients: ECOG‐ACRIN (E‐A) E2906 Randomized Study
Decitabine i.v. (20 mg/m^2^ for 5d q4w) vs. hydroxyurea p.o. (1‐4 g/d for 28d q4w)	NCT02214407	CMML	III	170	No difference in EFS (12.1 vs. 10.5 months) and OS (18.4 vs. 21.9 months) between the decitabine and hydroxyurea treatment.	N.a.	Itzykson M et al. JCO 2023: Decitabine Versus Hydroxyurea for Advanced Proliferative Chronic Myelomonocytic Leukemia: Results of a Randomized Phase III Trial Within the EMSCO Network
CC‐486 p.o. (300 mg for 14d q4w) vs. placebo	QUAZAR AML‐001 NCT01757535 (maintenance study)	AML in CR_(i)_ after IC	III	472	Augmented mOS (24.7 vs. 14.8 months) and RFS (10.2 vs. 4.8 months) in AML patients who achieved CR after IC and subsequently received CC‐486 compared to those receiving placebo.	FACIT and EQ‐5D‐3L—No differences in health‐ related QoL between the two cohorts.	Wei AH et al. NEJM 2020: Oral azacitidine maintenance therapy for acute myeloid leukemia in first remission
CC‐486 p.o.(300 mg for 21d q4w) vs. placebo	AZA‐MDS‐003 NCT01566695	Lower‐risk MDS	III	216	CC486 significantly enhanced RBC TI (31% vs. 11%) with prolonged mDoR (11.1 vs. 5.0 months) compared to placebo. Similarly, platelet recovery rate was improved with CC‐486 (24.3% vs. 6.5%).	FACIT and EQ‐5D‐3L—No differences in health‐ related QoL between the two cohorts.	Garcia‐Manero G et al. JCO 2021: Phase III, randomized, placebo‐controlled trial of CC‐486 (oral azacitidine) in patients with lower‐risk myelodysplastic syndromes
CC‐486 p.o. (200/300 mg for 7d q4w OR 150/200 mg for 14d q4w)	CC‐486‐AML‐002 NCT01835587 (post allo‐HCT maintenance)	MDS and AML after allo‐HCT	I/II	30	CC‐486 maintenance after allo‐HCT was well tolerated with limited AE grade ≥3. Pharmacokinetic analysis demonstrated that concomitant therapy after allo‐HCT did not alter CC‐486 pharmacokinetics. Estimated 1‐year survival rates were similar between the 7‐day and the 14‐day dosing cohorts (86% vs. 81%).	N.a.	De Lima, M et al. Transplantation and cellular therapy 2018: CC‐486 maintenance after stem cell transplantation in patients with acute myeloid leukemia or myelodysplastic syndromes
Guadecitabine s.c. (60/90 mg/m^2^ for 5d q4w OR 60 mg/m^2^ for 10d q4w)	NCT01261312	r/r AML	I/II	103	No difference in clinical benefit between the 5‐day course of 60 mg/m^2^ and 90 mg/m^2^ guadectiabine. Trend toward higher CR rates in patients receiving the 10‐day treatment compared to only the 5‐day treatment (30.2% vs. 16.0%).	N.a.	Roboz GJ et al. Cancer 2018: Dose, schedule, safety, and efficacy of guadecitabine in relapsed or refractory acute myeloid leukemia
Guadecitabine s.c. (60/90 mg/m^2^ for 5d q4w)	NCT01261312	Intermediate‐ or higher‐risk MDS and CMML	II	105	Similar ORR (40% vs. 55%) for the 60 mg/m^2^ and 90 mg/m^2^ cohorts with comparable adverse events.	N.a.	Garcia‐Manero G et al. Lancet Hematology 2019 Guadecitabine (SGI‐110) in patients with intermediate or high‐risk myelodysplastic syndromes: phase 2 results from a multicenter, open‐label, randomized, phase 1/2 trial
Guadecitabine s.c. (60 mg/m^2^ for 5d q4w) vs. TC (azacitidine s.c./i.v. for 7d q4w OR decitabine 20 mg/m^2^ for 5d q4w OR low dose Ara‐C)	ASTRIAL‐1 NCT02348489	AML	III	815	No statistically significant changes in mOS (7.1 vs. 8.5 months) and CR rates (19% vs. 17%).	N.a.	Fenaux P et al. Blood advances 2023: Guadecitabine vs. treatment choice in newly diagnosed acute myeloid leukemia: a global phase 3 randomized study
Guadecitabine s.c. (60 mg/m^2^ for 10d (cycle 1/2) followed for 5d (cycle 3+) q4w)	ASTRAL‐2 NCT02920008	r/r AML after IC	III	302	No statistically significant benefit in mOS (6.4 vs. 5.4 months) but higher rates of CR_(i)_ (27% vs. 14%) in guadecitabine cohort compared to the TC.	N.a.	Roboz GJ et al. Blood advances 2024: Guadecitabine vs. TC in relapsed/refractory AML after intensive chemotherapy: a randomized phase 3 ASTRAL‐2 trial
Cedazuridine/decitabine p.o. (100/35 mg) and decitabine (20 mg/m^2^ for 5d q4w) crossover study followed by cedazuridine/decitabine p.o. (100/35 mg) maintenance	NCT02103478	Intermediate‐ or higher‐risk MDS and CMML	II	80	Crossover study comparing p.o. decitabine/cedazuridine (cycle 1) followed by i.v. decitabine (cycle 2) or reverse, then followed by oral decitabine/cedazuridine maintenance. Similar mean decitabine systemic exposure (measured as oral/i.v. ratios of geometric LSM 5‐day AUC) with dose confirmation (93.5%) versus fixed‐dose combination (97.6%). Less than 1% difference in DNA methylation between the two interventions. 60% ORR.	N.a.	Garcia‐Manero G et al. Blood advances 2020: Oral cedazuridine/Decitabine for MDS and CMML: a phase 2 pharmacokinetc/pharmcodynamic randomized crossover study
Cedazuridine/decitabine p.o. (100/35 mg) and decitabine (20 mg/m^2^ for 5d q4w) crossover study followed by cedazuridine/decitabine p.o. (100/35 mg) maintenance	ASCERTAIN NCT03306264	MDS, AML and CMML	III	138	Crossover study comparing p.o. decitabine/cedazuridine (cycle 1) followed by i.v. dectiabine (cycle 2) or reverse, then followed by oral decitabine/cedazuridine maintenance. Similar pharmacokinetics in AML/MDS/CMML patients receiving fixed drug combination of decitabine/cedazuridine versus decitabine (98% vs. 93%). Similar safety profile between the two interventions.	N.a.	Garcia‐Manero G et al. Lancet Hematology 2024: Oral decitabine–cedazuridine versus intravenous decitabine for myelodysplastic syndromes and chronic myelomonocytic leukaemia (ASCERTAIN): a registrational, randomized, crossover, pharmacokinetics, phase 3 study
Cedazuridine/decitabine p.o. (100/35 mg) and decitabine (20 mg/m^2^ for 5d q4w) crossover study followed by cedazuridine/decitabine p.o. (100/35 mg) maintenance	ASTX727‐02 NCT03306264	AML	III	87	Crossover study comparing p.o. decitabine/cedazuridine (cycle 1) followed by i.v. decitabine (cycle 2) or reverse, then followed by oral decitabine/cedazuridine maintenance. Similar systemic decitabine exposure (5‐day AUC ratio 99.64) and methylation rates (≤1.1% difference).	N.a.	Geissler K et al. Br J Hematology 2024: Oral decitabine/cedazuridine versus intravenous decitabine for acute myeloid leukemia: a randomized, crossover, registration, pharmacokinetics study
Decitabine i.v. (20 mg/m^2^ for 3d q4w) vs. azacitidine i.v./s.c. (75 mg/m^2^ for 3d q4w)	NCT01720225	Lower‐risk MDS	II	113	Significantly improved ORR (67% vs. 48%) in the decitabine compared to the azacitidine cohort. Among the 59 patients who were transfusion‐depend at baseline, 32% achieved TI, however decitabine demonstrated notably greater efficacy in achieving transfusion independence compared to azacitidine (41% vs. 15%).	N.a.	Sasaki K et al. NEJM Evid 2022: Low‐dose decitabine versus low‐dose azacitidine in lower‐risk MDS

Abbreviations: allo‐HCT, allogeneic hematopoietic stem cell transplantation; AML, acute myeloid leukemia; AMLFS, acute myeloid leukemia‐free survial; Ara‐C, cytarabine; AUC, area under the curve; BSC, best supportive care; (c)CR, (composite) complete remission; CCR, conventional care regimens; CMML, chronic myelomonocytic leukemia; CR_(i)_, complete remssion _(incomplete)_; DFS, disease‐free survival; EORTC QLQ‐30, European Organisation for Research and Treatment of Cancer quality of life questionnaire 30; FACIT, Functional Assessment of Chronic Illness Therapy questionnaire; HRQoL, health related quality of life; i.v., intravenous; IC, intensive chemotherapy; LSM, least‐square means; (m)DoR, (median) duration of response; (m)EFS, (median) event‐free survival; (m)OS, (median) overall survival; MDS, myelodysplastic syndrome; MHI, mental health inventory (questionnaire); MPN, myeloproliferative neoplasms; MRD, minimal residual disease; N.a., not applicable; ORR, overall response rate; p.o., per os; PFS, progression‐free survival; r/r, relapsed or refractory; RBC, red blood cells; RFS, relapse‐free survival; s.c., subcutaneous; TC, treatment of choice; TI, transfusion independence.

AML and MDS are clonal hematological diseases characterized by aberrant proliferation and disturbed differentiation of myeloid cells, often driven by critical molecular alterations such as hypermethylation events.^32^, ^33^ To counteract these observed changes, the two first‐generation DNA methyltransferase inhibitors (DNMTi) azacitidine and decitabine (5‐aza‐2′‐deoxycytidine, 5azaCdR) have been clinically developed.^13^ Although not initially recognized as an epigenetic drug, early preclinical studies in cell culture and mouse models demonstrated their high potency, despite significant side effects.^10^ In the 1970s, azacitidine was introduced as a treatment for childhood and adult leukemia in North America and Europe.^34^, ^35^ Despite encouraging results, its usage was limited by severe myelosuppression and gastrointestinal (GI) toxicity, which can be primarily attributed to the high doses used in these early studies.

Azacitidine gained approval by the U.S. Food and Drug Administration (FDA) for the treatment of MDS in 2004, following the results of the above‐mentioned National Cancer Institute (NCI)‐sponsored phase III clinical trial conducted by the CALGB, along with two supportive phase II CALGB studies.^36^, ^37^, ^38^ The phase III trial, chaired by Dr. Lewis Silverman and published in 2002,^29^ involved 191 patients with MDS, demonstrating a 60% disease control rate in the azacitidine arm compared to only 5% in the best supportive care (BSC) arm. This study also established the standard dose of subcutaneous azacitidine as 75 mg/m^2^ for 7 days in a 28‐day cycle. In 2007, the FDA expanded its approval to include intravenous administration based on an uncontrolled phase II study, with pharmacokinetic bioavailability data.^39^ By 2008, the European Medicines Agency (EMA) had approved azacitidine for higher‐risk MDS patients based on the results of the Aza‐001 trial. This multicenter, open‐label randomized phase III trial included 358 patients who received either a 7‐day treatment of 75 mg/m^2^ subcutaneous azacitidine for at least six cycles or conventional care, which included BSC, low dose cytarabine (20 mg/m^2^) or IC, consisting of cytarabine in combination with an anthracycline.^40^, ^41^ After a median follow‐up of 21.1 months, the study met its primary endpoint, with an overall survival (OS) of 24.5 months in the azacitidine arm, compared to 15.0 months for the conventional care arm (HR 0.58, 95% CI 0.43–0.77, *p* = 0.0001).

#### 1.2.2 Azacitidine in AML

In 2015, the applicability of azacitidine was further evaluated in medically non‐fit AML patients with at least 30% blasts in an international, multicenter, open‐label phase III trial also comparing azacitidine to conventional care regimens (CCR).^42^ This study, which included 488 patients assigned 1:1 to both interventions, demonstrated improved OS in the azacitidine arm of 10.4 months (95% CI 8.0–12.7), compared to the 6.5 months in the CCR arm (95% CI 5.0–8.6). Concurrently, efforts have been made to enhance treatment efficacy and durability by incorporating azacitidine into standard IC regimens in fit older AML patients. However, the phase II AML‐AZA trial of the Study Alliance Leukemia (SAL) German Cooperative Group, which enrolled 214 patients, failed to demonstrate an additional benefit of azacitidine and IC over IC alone in event‐free survival (EFS), while simultaneously leading to a marked increase in treatment‐related toxicities.^43^ Most recently, azacitidine has also been evaluated as a maintenance therapy in AML patients who achieved complete remission (CR) after IC. The HOVON97 trial demonstrated the superiority of an azacitidine maintenance (50 mg/m^2^ for 7 days in a 28‐day cycle) compared to observation at 12 months disease‐free survival (DFS, 64% vs. 42%, *p* = 0.04); OS, however, remained unaffected.^44^

#### 1.2.3 Oral azacitidine (CC‐486)

In recent years, an oral azacitidine formulation (also referred to as CC‐486) has been granted approval by the FDA and EMA based on the results of the QUAZAR AML‐001 trial, which investigated CC‐486 as a maintenance therapy for older AML patients in first remission after IC.^45^ The trial randomized 472 patients to receive either 14 days of 300 mg oral azacitidine once daily or placebo, repeated every 28 days. The primary endpoint of median OS was met, with an OS of 24.7 months in the oral azacitidine cohort compared to 14.8 months in the placebo cohort (*p* < 0.001). The secondary endpoint of relapse‐free survival (RFS) was also met. Side effects of CC‐486 were overall comparable to those of the subcutaneous and intravenous administration, mainly being grade I‐II (GI) side effects (more pronounced than with parenteral administration) and grade III‐IV hematological side effects.

In addition, the effects of CC‐486 on transfusion dependency in lower‐risk MDS patients have been evaluated.^46^ The AZA‐MDS‐003 trial assigned a total of 216 patients to receive either CC‐486 or placebo. The primary endpoint of red blood cell (RBC) transfusion independence (TI) for at least 56 days was reached in 30.8% of patients receiving CC‐486 compared to 11.1% in the respective placebo cohort (OR 3.6, 95% CI 1.7–7.4, *p* = 0.0002). Likewise, 24.3% of patients receiving CC‐486 achieved improvement in platelet counts compared to 6.5% in the placebo arm.

#### 1.2.4 Decitabine in MDS

In parallel with the development of azacitidine, the development of decitabine, the chemically very closely related deoxyribonucleoside and first generation DNMTi, has also been steadily pursued as a less‐intensive alternative to standard IC in patients with myeloid malignancies. Similar to azacitidine, decitabine was first synthesized in the 1960s, with initial phase I clinical data as a traditional chemotherapeutic being discouraging due to its high toxicities.^47^ However, the repurposing of decitabine, an azanucleoside drug that is incorporated solely into DNA, with adjusted dosage regimens, has led to its resurgence. It was investigated both in the US and in Europe (particularly in Italy, the Netherlands and Germany), and in the early 1990s, the MD Anderson Cancer Center started incorporating decitabine in their clinical trials. In 2006, Kantarjian et al. first reported results of a randomized controlled phase III clinical trial in MDS patients.^48^ In this trial, 170 MDS patients were randomized to receive either 15 mg/m^2^ intravenous decitabine every 8 h for a total of 3 days, repeated every 6 weeks, or BSC. The trial met its primary endpoint of increased overall response rate (ORR), with 17% in the decitabine group vs. 0% in the BSC group (*p* < 0.001) and a trend toward longer time to progression to AML or death. Based on these trials, the FDA approved decitabine in 2006 for the treatment of higher‐risk MDS.

These results were confirmed by a European EORTC (European Organisation for the Research and Treatment of Cancer) Leukemia Cooperative Group phase III clinical trial assessing decitabine versus BSC in older intermediate‐ or higher‐risk MDS patients.^49^ Decitabine, while significantly prolonging progression‐free survival (PFS) from 3.3 to 6.0 months (HR 0.68; 95% CI, 0.52–0.88; *p* = 0.004), did not yield significantly prolonged OS (10.1 months in the decitabine cohort versus 8.5 months in the BSC arm).

#### 1.2.5 Decitabine in AML: non‐fit patients

In 2012, the DACO‐016 trial, a multicenter, open‐label phase III trial followed, which investigated the effect of decitabine in newly diagnosed, medically non‐fit AML patients.^50^ The study did not initially detect an increase in OS with decitabine (7.7 months, 95% CI 6.5–9.2) that reached statistical significance compared to treatment of choice (TC, 5.0 months, 95% CI 4.3–6.3), which was, however, reached in a second OS analysis. The CR(p) rates of patients treated with decitabine were significantly higher (17.8%) compared to the TC cohort (7.8%, OR 2.5, 95% CI 1.4–4.8, *p* = 0.001). This study also established the benchmark dosing regimen of 20 mg/m^2^ intravenous decitabine daily for 5 days, repeated every 4 weeks.

A randomized study conducted at the MD Anderson Cancer Center addressed the question of the value of decitabine dose intensification in medically non‐fit elderly AML patients, comparing the 10‐day to the 5‐day treatment schedule. In a phase II clinical trial involving 71 patients with newly diagnosed AML, Short et al. reported similar clinical CCR rates (43% vs. 40%) as well as 1‐year OS outcomes (25% vs. 25%) between the 5‐day and 10‐day decitabine treatment groups.^51^ However, the validity of these findings has been called into question due to the study's limited statistical power, leaving the equivalence between the two regimens unresolved, particularly in these elderly and non‐fit patients.^52^

#### 1.2.6 Decitabine in AML: fit patients

The EORTC Leukemia Cooperative Group, the Gruppo Italiano Malattie EMatologiche dell 'Adulto (GIMEMA) and German MDS Study Group subsequently conducted the Intergroup trial AML21, which compared standard “7 + 3” intensive induction combination chemotherapy with 10‐day decitabine monotherapy followed by allogeneic hematopoietic stem cell transplantation (allo‐HCT).^53^, ^54^ This first trial to investigating a de‐escalation approach versus intensive induction in a large, multinational phase III European trial randomized 606 patients in a 1:1 ratio and demonstrated equivalence in 4‐year OS (26% vs. 30%, HR 1.04, 95% CI 0.86–1.26, *p* = 0.68) between the DNMTi and IC treatments. Of note, grade ≥3 adverse events (AE) were significantly less frequent in the decitabine cohort, highlighting its potential for reduced treatment‐related toxicity. Importantly, health‐related quality of life (QoL) in the decitabine arm was less compromised than with IC, a result which, while very much intuitive, had not been demonstrated before in a randomized trial. This result had major implications for the use of DNMTi‐based “bridging” therapy of AML/MDS patients to the curative approach of allo‐HCT, as patients having received less toxic pre‐treatment (by the principle of “less is more”) may have a more favorable transplant outcome than those having received repetitive cycles of standard chemotherapy.

#### 1.2.7 Decitabine in AML: maintenance treatment

The ECOG‐ACRIN E2906 trial led by Foran et al. confirmed the significance of a decitabine maintenance after IC in an older AML patient population.^55^ In this trial, a total of 120 patients were randomized 1:1 to a 3‐day course of decitabine maintenance schedule (20 mg/m^2^, repeated every 4–6 weeks), first reported by Lübbert et al.,^56^ or observation only. Median OS was significantly improved in the decitabine cohort compared to observation only (HR 0.69, 80% CI 0.51–0.93, *p* = 0.06) with DFS indicating a trend toward favoring decitabine as well, although it is worth noting that the authors set a rather unusual significance level at 0.1, with an 80% CI.

#### 1.2.8 Decitabine in chronic myelomonocytic leukemia (CMML)

Interestingly, decitabine's therapeutic potential has also been investigated in chronic myelomonocytic leukemia (CMML), a hematological disease that has often been included in traditional DNMTi trials due to its risk of progression to AML but rarely been studied on its own. In 2023, a phase III European Myelodysplastic Neoplasms Cooperation Group trial was reported that assigned 170 CMML patients to a 5‐day decitabine treatment versus BSC including hydroxyurea.^57^ While the study failed to demonstrate superiority of decitabine as regards EFS and OS, the risk of CMML progression or transformation to AML was significantly reduced in the decitabine compared to the hydroxyurea cohort (HR 0.62, 95% CI 0.41–0.94, *p* = 0.005).

#### 1.2.9 Azacitidine versus decitabine: prospective comparative studies

Until now, most clinical investigators have chosen to investigate either azacitidine or decitabine, with only very few directly comparing these two therapies.^53^ Among the very few notable studies is a randomized phase II clinical trial that assigned 113 lower‐risk MDS patients to standard dose azacitidine and decitabine in order to compare transfusion dependency.^58^ Out of 59 patients' transfusion‐dependency at baseline, a significantly higher proportion reached TI in the decitabine versus azacitidine cohort (41% vs. 15%, *p* = 0.0039), suggesting decitabine being superior in this head‐to‐head comparison. In another randomized trial, Jabbour et al. compared responses of a 3‐day decitabine treatment to a 3‐day azacitidine treatment in 113 lower‐risk MDS and MDS/MPN (myelodysplastic syndrome/myeloproliferative neoplasms) overlap patients.^59^ While the ORR was significantly higher in the decitabine arm (70% vs. 49%), this was not reflected by median EFS nor transfusion independency.

#### 1.2.10 Guadecitabine (SGI‐110)

Guadecitabine (SGI‐110), a second generation DNMTi, is a dinucleotide that acts as a prodrug of decitabine. Guadecitabine has a longer half‐life compared to decitabine, potentially enhancing its hypomethylating activity.^60^ Initial data from phase II trials in both AML and high‐risk MDS demonstrated good feasibility of guadecitabine with similar ORR and CR rates between the two investigated doses of 60 mg/m^2^ and 90 mg/m^2^.^61^, ^62^ However, in a large randomized phase III trial of guadecitabine vs. investigator's choice in newly diagnosed AML, the trial could not demonstrate superiority in OS (7.1 vs. 8.5 months) nor CR rate (19% vs. 17%) for a guadecitabine treatment regimen of 60 mg/m^2^ over 5 days in comparison to TC.^63^ However, the phase III ASTRAL‐2 trial (in relapsed/refractory [r/r] AML patients) which investigated a longer, 10‐day course of 60 mg/m^2^ guadecitabine, reported higher CCR rates in the guadecitabine arm (27% vs. 14%, *p* < 0.01) than the TC, albeit without affecting OS.^64^

#### 1.2.11 Oral decitabine formulation (ASTX727)

Similar to CC‐486, an oral formulation of decitabine (ASTX727) has been developed to facilitate at‐home administration. This was with the goal of improving patients' QoL by limiting time spent in the hospital, therefore reducing the “time toxicity” that can burden non‐curative treatment conducted with parenteral drug administration. The most promising approach involves the oral formulation of the combination of decitabine with cedazuridine, a small‐molecule inhibitor of cytidine deaminase that limits the degradation of oral decitabine. This fixed‐dose combination, marketed as ASTX727, was compared to i.v. decitabine in the ASCERTAIN trial, a multicenter, randomized, open‐label, crossover pharmacokinetic study in adults with intermediate‐ or higher‐risk MDS and CMML.^65^, ^66^ The trial demonstrated pharmacological and pharmacodynamical equivalence to i.v. decitabine, leading to FDA approval for MDS, as this oral formulation could provide patients with a more convenient and equally effective treatment option. These results have also been validated in another crossover phase III study, conducted in Europe, comparing, in newly diagnosed AML patients, ASTX727 with i.v. decitabine and indicating similar systemic exposure and demethylation rates,^67^ resulting in the EMA approval of ASTX727 in newly diagnosed, IC ineligible AML patients in 2023.

In addition to the currently approved hypomethylating agents, a promising alternative class of direct enzymatic DNMT1i are emerging.^68^, ^69^ GSK3685032 is a first‐in‐class, reversible DNMT1i that has shown preclinical efficacy by inducing loss of DNA methylation, transcriptional activation and cancer cell growth inhibition in various cancer cell lines, however clinical data is still lacking.^68^ In r/r MDS/AML, the novel oral DNMT1i inhibitor NTX‐301 is actively tested in a phase I trial (NCT04167917) to assess the safety and tolerability as well as the dose‐limiting toxicities.^69^ This trial is expected to complete in 2027 and will include the secondary endpoint of ORR, PFS and OS. These novel developments underscore the translational potential of enzymatic DNMT1‐targeted therapy as a more selective and potentially less toxic alternative to traditional hypomethylating agents, marking an important future direction in epigenetic treatment modalities.

### 1.3 Demethylating agents and cellular therapies in MDS and AML

#### 1.3.1 Decitabine conditioning for allogeneic stem cell transplantation

A Chinese phase III trial evaluated the impact of a modified conditioning regimen for allo‐HCT, incorporating decitabine as well as G‐CSF in addition to standard busulfan and cyclophosphamide in patients with relapsed MDS or secondary AML.^70^ The study enrolled 202 patients across six Chinese sites and demonstrated a significant reduction in 2‐year cumulative incidence of relapse in the decitabine and G‐CSF augmented treatment arm compared to standard busulfan and cyclophosphamide conditioning (10.9% vs. 24.8%, HR 0.39, 95% CI 0.19–0.79, *p* = 0.011). Notably, the cumulative day 100 post allo‐HCT incidences of infections, GI side effects, and death were comparable between the two cohorts, thereby suggesting broader applicability of this modified conditioning.

#### 1.3.2 Azacitidine maintenance after transplant

While both AML and MDS are potentially curable diseases after allo‐HCT, relapse remains one of the most significant causes of death in these patients.^71^ As a result, DNMTi maintenance and relapse treatment strategies have been explored, with the rationale of both enhancing the Graft‐versus‐Leukemia (GvL) effect and instigating direct antitumor effects. Initial evidence supporting the use of low‐dose azacitidine as a maintenance or salvage therapy post allo‐HCT emerged around 2009.^72^ In this early trial, Jabbour et al. reported an actuarial 1‐year EFS of 55% as well as an OS of 90% with low‐dose azacitidine. However, this study was limited by a small cohort of 17 patients and a median follow‐up of only 16 months after allo‐HCT and 11 months post‐azacitidine treatment. A subsequent, larger follow‐up trial of 45 patients who received low‐dose azacitidine as a maintenance therapy after allo‐HCT in heavily pretreated MDS/AML cases demonstrated an EFS of 58% at 18.2 months (95% CI 11.9–NA) and OS of 77% at a median of 30.8 months (95% CI 14.3–NA), indicating improved clinical outcomes.^73^

#### 1.3.3 CC‐486 maintenance after transplant

Additionally, the oral azacitidine formulation CC‐486 has been evaluated as a maintenance therapy after allo‐HCT in MDS/AML patients, demonstrating a feasible toxicity profile and low rates of relapse and Graft‐versus‐Host Disease (GvHD).^74^ Mechanistic studies have also suggested that azacitidine can reinitiate the expression of silenced leukemia antigen genes and increase the frequencies of regulatory T cells, thereby further enhancing GvL and GvHD effects.^75^, ^76^ These findings prompted the initiation of a phase III clinical trial. However, an open‐label, randomized phase III clinical trial enrolling 187 higher‐risk MDS/AML patients to compare azacitidine maintenance versus observation after allo‐HCT failed to demonstrate differences in RFS (2.07 vs. 1.28 years) and OS (2.52 vs. 2.56 years).^77^ In this context, another phase III clinical trial comparing CC‐486 maintenance versus placebo in MDS/AML patients after allo‐HCT is expected to finish recruiting in 2025 (AMADEUS, NCT04173533).

#### 1.3.4 Decitabine maintenance after transplant

Maintenance therapy with decitabine following allo‐HCT in MDS/AML patients has also been exploited. A phase I, dose‐escalating trial, indicating manageable toxicities,^78^ as well as a currently ongoing phase Ib clinical trial investigating the combination of oral decitabine and cedazuridine as maintenance after allo‐HCT for MDS, are currently ongoing, with results anticipated soon (NCT04980404). However, larger, especially phase II/III clinical trials for decitabine maintenance after allo‐HCT are still lacking.

#### 1.3.5 MRD‐driven relapse treatment after transplant: RELAZA trials

In AML and MDS patients who displayed features of imminent relapse after allo‐HCT, indicated by measurable residual disease (MRD) positivity, the phase II RELAZA‐1 trial demonstrated the feasibility of azacitidine with an ORR of restored or stable donor chimerism of 80% and a subsequently delayed relapse at a median of 231 days.^79^ The follow‐up RELAZA‐2 trial confirmed these results, investigating 60 high‐risk MDS or AML patients who became MRD‐positive after IC or allo‐HCT and were subsequently eligible for an azacitidine salvage treatment. Patients who remained MRD positive had a significantly lower 12‐month RFS at 46% in comparison to the 88% seen in MRD‐negative converters (HR 6.6, 95% CI 3.7–11.8, *p* < 0.0001).^80^

#### 1.3.6 Combination of azacitidine or decitabine with donor‐lymphocyte infusions

For patients experiencing relapse of MDS/AML, combination treatment of donor lymphocyte infusions (DLI) and DNMTi has been investigated. A pilot study by Lübbert, Wäsch et al. demonstrated the feasibility of combining DLI with a 3‐day 100 mg (flat dose) subcutaneous azacitidine in relapsed AML patients, even within an adverse cytogenetic population.^81^ A subsequent single‐arm phase II clinical trial investigating the effects of DLI and azacitidine in r/r MDS/AML followed shortly thereafter, demonstrating an ORR of 30%.^82^ These results were later confirmed by an even larger retrospective analysis of 154 post‐allo‐HCT relapsed MDS/AML patients demonstrating an ORR of 33% (27% CR, 6% partial remission [PR]) and a 2‐year OS of 29%.^83^ Similarly, a retrospective analysis of 36 patients receiving DLI and decitabine post allo‐HCT relapse showed a similar efficacy with an ORR of 25%.^84^ However, the 2‐year OS was significantly lower at 11%, thereby suggesting DLI and decitabine may be more feasible as a second‐line treatment after azacitidine failure. Most recently, novel dual treatment combinations have been under investigation.

#### 1.3.7 DNMTi and nivolumab in AML patients relapsing after transplant: the NIFAR trial

A prospective phase II clinical trial (EudraCT‐No. 2017–002194‐18) combining azacitidine and the anti‐PD1 inhibitor nivolumab in relapsed AML patients after allo‐HCT demonstrated an ORR of 25% (12.5% CR_(i)_, 12.5% PR) and a median survival of 15.6 months.^85^ This response rate was attributed to higher frequencies of activated, non‐quiescent CD8^+^ T cells as well as a shift toward pro‐inflammatory expression profiles of T and myeloid cells.

#### 1.3.8 DNMTi and CAR‐T cell therapies in AML

Besides the combination with immune checkpoint inhibitors for the treatment of AML, DNMTi were also combined with chimeric antigen receptor (CAR)‐T cells directed against CD123 to treat AML in mice.^86^ These studies used anti‐CD123 CAR‐T cells with a CD28‐OX40‐CD3ζ intracellular signaling domain. Interestingly, azacitidine treatment enhanced CAR‐T cell‐mediated AML blast elimination by increasing CD123 expression on leukemia cells. In addition, leukemia‐bearing mice treated with azacitidine displayed lower frequencies of exhausted cytotoxic CTLA‐4^negative^ anti‐CD123 CAR‐T cells phenotype with higher TNFα production.^86^ Building on these promising preclinical data, clinical trials assessing the synergistic effects of DNMTi and CAR‐T therapies in AML patients are still to be awaited.

### 1.4 DNMTi‐based combination studies in MDS and AML

#### 1.4.1 DNMTi in combination with BCL‐2 inhibition

DNMTi have been extensively studied in various combination therapies, primarily as doublets, in frail patients or those for whom IC is not suitable (Table 2 summarizes combination therapy randomized clinical trials; Table 3 summarizes all FDA/EMA approved single/combination therapies). Combination therapies in both newly diagnosed and r/r AML/MDS patients have become an area of intensive study, especially with an increasing number of novel therapeutics becoming available and possible synergistic effects of dual treatment.^13^, ^87^ Among the most promising combination therapies are DNMTi in combination with inhibitors of the antiapoptotic protein BCL‐2, such as venetoclax. (Pre)‐clinical data suggest that these agents synergistically enhance each other's effect, offering a more potent therapeutic approach than either agent alone.^88^, ^89^ For instance, azacitidine was shown to reduce levels of antiapoptotic MCL‐1 in AML blasts and induce an integrated stress response via PAIMP1 induction, making blasts susceptible to venetoclax‐induced apoptosis.^90^ Conversely, venetoclax inhibits dihydroorotate dehydrogenase, a key enzyme in conferring resistance to DNMTi.^91^ Additional mechanisms, such as selective alteration of the citric acid cycle energy metabolism in leukemia stem cells (LSCs), inhibiting cellular energy supply, are discussed and may further contribute to the efficacy of this combination treatment.^92^ Common side effects of this dual treatment include myelosuppression, infections, and GI symptoms.^93^

**TABLE 2:** Clinical phase II/III trial overview of DNMTi‐based combination therapies in hematological malignancies.

Drugs and dosages/control arm	Study name and trial number	Entity	Phase	Number of patients	Results and outcome	Quality of life	Citation
Azacitidine s.c./i.v. (75 mg/m^2^ for 7d q4w) + venetoclax p.o. (400 mg with ramp up for 28d q4w) vs. azacitidine s.c./i.v. + placebo p.o.	VIALE‐A NCT02993523	AML	III	431	Improved mOS (14.7 vs. 9.6 months) and cCR rate (66.4% vs. 28.3%) in the azacitidine + venetoclax cohort compared to the azacitidine + placebo control.	PROMIS Fatigue SF7a and EORTIC QLQ‐30—No differences between the two cohorts were reported.	DiNardo CD et al. NEJM 2020: Azacitidine and venetoclax in previously untreated acute myeloid leukemia
Azacitidine s.c./i.v. (75 mg/m^2^ for 7d q4w) + venetoclax p.o. (400 mg with ramp up for 28d q4w) vs. azacitidine s.c./i.v. + placebo p.o.	VERONA NCT04401748	Higher‐risk MDS	III	Ongoing, currently approximately 500 patients enrolled	Negative trial but publication still pending. Primary endpoint will be overall survival, secondary endpoints include modified ORR, hematological improvement, TI, QoL among others. (Results expected end of 2025/early 2026).	Expected to be reported using PROMIS Fatigue SF7a and EORTIC QLQ‐30.	N.a.
Decitabine i.v. (20 mg/m^2^ for 5d q4w) OR decitabine i.v. (20 mg/m^2^ for 5d q4w) + valproate p.o. (d6—end of treatment) OR decitabine i.v. (20 mg/m^2^ for 5d q4w) + ATRA p.o.(45 mg/m^2^ d6‐28 q4w) OR decitabine i.v. (20 mg/m^2^ for 5d q4w) + valproate p.o. (d6‐end of treatment) + ATRA p.o.(45 mg/m^2^ d6‐28 q4w)	DECIDER (I) NCT00867672	AML	II	200	Augmented mOS (8.2 vs. 5.1 months) and ORR (21.9% vs. 13.5%) in the decitabine + ATRA cohort compared to decitabine only. No differences in mOS and ORR reported with the addition of valproate to decitabine ± ATRA.	EORTIC QLQ‐30—No difference in outcome with the addition of ATRA or valproate were reported.	Lübbert M et al. JCO 2020: Valproate and retinoic acid in combination with decitabine in elderly nonfit patients with acute myeloid leukemia: results of a multicenter, randomized, 2 × 2, phase II trial
Azacitidine s.c./i.v. (75 mg/m^2^ for 7d q4w) + pracinostat p.o. (60 mg tiw for 21d q4w) vs. azacitidine s.c./i.v. + placebo p.o.	PRIMULA NCT03151408	AML	III	406	No significant difference in mOS (9.95 months in both) nor CCR (36.0% vs. 31.5%) between the interventions and control cohort.	N.a.	Garcia‐Manero G et al. Leukemia Research 2024: Pracinostat combined with azacitidine in newly diagnosed adult acute myeloid leukemia (AML) patients unfit for standard induction chemotherapy: PRIMULA phase III study
Azacitidine s.c./i.v. (75 mg/m^2^ for 7d q4w) + pracinostat p.o. (60 mg tiw for 21d q4w) vs. azacitidine s.c./i.v. + placebo p.o.	NCT01873703	Higher‐risk MDS	II	102	No significant differences between the pracinostat and placebo cohort in OS (16 vs. 19 months), PFS (11 vs. 9 months) and CR rates by cycle 6 (18% vs. 33%).	N.a.	Garcia‐Manero G et al. “Cancer 2017: Phase 2, randomized, double‐blind study of pracinostat in combination with azacitidine in patients with untreated, higher‐risk myelodysplastic syndromes.” *Cancer* 123.6 (2017): 994–1002
Azacitidine s.c. (50 mg/m^2^ for 10d q4w) + entinostat p.o. (4 mg/kg/d on d3,10 q4w) vs. azacitidine s.c.	NCT00313586	MDS (all subtypes), AML and CMML	II	149	No significant difference in hematological normalization (32% vs. 27%) nor hematological ORR (46% vs. 44%) between the intervention and control cohort.	N.a.	Prebet T et al. JCO 2014: Prolonged administration of azacitidine with or without entinostat for myelodysplastic syndrome and acute myeloid leukemia with myelodysplasia‐related changes: results of the US Leukemia Intergroup trial E1905
Azacitidine s.c./i.v. (75 mg/m^2^ for 7d q4w) + ivosidenib p.o. (500 mg for 28d q4w) vs. azacitidine s.c./i.v. + placebo p.o.	NCT03173248	IDH1^mut^ AML	III	146	Azacitidine + ivosidenib augmented EFS (0.33) and mOS (24.0 vs. 7.9 months) in IDH1^mut^ AML compared to azacitidine + placebo.	EORTIC QLQ‐30—No difference at baseline between the two cohorts, however results favored the azacitidine + ivosidenib cohort after an initial decline during treatment initiation.	Montesinos P et al. NEJM 2022: Ivosidenib and azacitidine in IDH1‐mutated acute myeloid leukemia
Azacitidine s.c./i.v.(75 mg/m^2^ for 7d q4w) + gilteritinib p.o. (120 mg for 28d q4w) vs. azacitidine s.c./i.v.	LACEWING NCT02752035	FLT3^mut^ AML	III	123 patients enrolled at interim analysis	Slight improvement in mOS in FLT3mut AML patients receiving azacitine + gilteritinib arm compared to the azacitidine only (9.82 vs. 8.87 months). However, the study was terminated based on previously set protocol specific boundaries.	N.a.	Wang ES et al. Blood 2022: Phase 3 trial of gilteritinib plus azacitidine vs. azacitidine for newly diagnosed FLT3 mut + AML ineligible for intensive chemotherapy
Azacitidine s.c/i.v.(75 mg/m^2^ for 7d q4w) + eprenetapopt p.o. (4500 mg for 4d q4w)	NCT03745716	TP53^mut^ MDS and oligoblastic AML (20–30% blasts)	Ib/II	55	ORR of 71% and CR rate of 44% in TP53^mut^ AML/MDS. ORR (73% vs. 64%) and CR rates (58% vs. 36%) were higher in MDS compared to AML. mOS was 10.8 months.	N.a.	Sallmann DA et al. JCO 2021: Eprenetapopt (APR‐246) and azacitidine in TP53‐mutant myelodysplastic syndromes
Decitabine i.v. (20 mg/m^2^ for 5d q4w) + ibrutinib p.o. (560 mg for 18/23d begun after decitabine q4w) vs. decitabine i.v.	HOVON135/SAKK15/30 NL5751/2015–002855‐85	Higher‐risk MDS and AML	II	144	No significant differences in mOS (11.0 vs. 11.5 months) nor CCR (41% vs. 50%) between the decitabine + ibrutinib and the decitabine only cohort.	N.a.	Huls G et al. Blood advances 2020: Ibrutinib added to 10‐day decitabine for older patients with AML and higher risk MDS
Decitabine i.v. (20 mg/m^2^ for 5d q4w) + bortezomib s.c. (1,3 mg/m^2^ d1,4,8,11 q4w) vs. decitabine i.v.	CALGB11002 NCT01420926	AML (excluding FLT3^mut^ and good risk leukemia)	II	165	No significant difference in mOS (8.9 vs. 9.3 months) nor ORR (38% vs. 39%) or DoR (15.3 vs. 10.9 months) between the decitabine + bortezomib and decitabine only cohort.	N.a.	Roboz GJ et al. Blood advances 2018: Randomized trial of 10 days of decitabine±bortezomib in untreated older patients with AML: CALGB 11002 (Alliance)
Azacitidine s.c./i.v.(75 mg/m^2^ for 7d q4w) OR azacitidine s.c./i.v.(75 mg/m^2^ for 7d q4w) + lenalidomide p.o. (10 mg for 21d q4w) OR azacitidine s.c./i.v.(75 mg/m^2^ for 7d q4w) + vorinostat (300 mg bid d3‐9 q4w)	North American Intergroup Study 1117 NCT01522976	Higher‐risk MDS and CMML	II/III	277	Improved ORR (49% vs. 38%) with azacitidine + lenalidomid compared to azacitidine only.	N.a.	Sekeres MA et al. JCO 2017: Randomized phase II study of azacitidine alone or in combination with lenalidomide or with vorinostat in higher‐risk myelodysplastic syndromes and chronic myelomonocytic leukemia: North American Intergroup Study SWOG S1117
Azacitidine s.c./i.v. (75 mg/m^2^ for 7d q4w) + pevonedistat i.v. (20 mg/m^2^ on d1,3,5 q4w) vs. azacitidine s.c./i.v.	NCT02610777	Higher‐risk MDS, oligoblastic AML (20–30% blasts) and CMML	II	120	Azacitidine + pevonedistat demonstrated improved EFS (20.2 vs. 14.8 months) as well as increased ORR (79% vs. 57%) compared to single agent azacitidine treatment in higher‐risk MDS.	EORTIC QLQ‐30, QOL‐ONE and EQ‐5D‐5L—No significant changes between the two cohorts.	Sekeres, M, et al. Leukemia 2021: Randomized phase 2 trial of pevonedistat plus azacitidine versus azacitidine for higher‐risk MDS/CMML or low‐blast AML
Azacitidine s.c./i.v. (75 mg/m^2^ for 7d q4w) + pevonedistat i.v. (20 mg/m^2^ on d1,3,5 q4w) vs. azacitidine s.c./i.v.	PANTHER NCT03268954	Higher‐risk MDS, oligoblastic AML (20–30% blasts) and CMML	III	454	Azacitidine + pevonedistat failed to demonstrate superiority for the primary end point of EFS (17.7 vs. 15.7 months) in comparison to azacitidine treatment only. No difference in mOS in the MDS cohort (21.6 vs. 17.2 months) nor AML cohort (14.5 vs. 14.7 months) between the dual versus single agent treatment.	N.a.	Adès, L et al. Blood advances 2022: Pevonedistat plus azacitidine vs. azacitidine alone in higher‐risk MDS/chronic myelomonocytic leukemia or low‐blast‐percentage AML
Azacitidine s.c./i.v. (75 mg/m^2^ for 7d q4w) + magrolimab i.v. (30 mg/kg with ramp up on d1, 4,8,11,15, followed by q1w for 5 weeks and subsequently q2w administration q4w) vs. azacitidine s.c./i.v. + placebo i.v.	ENHANCE NCT04313881	Intermediate−/higher‐risk MDS	III	539	Terminated due to futility. Primary outcomes of CR and OS have not yet been reported.	N.a.	N.a.
Azacitidine s.c./i.v. (75 mg/m^2^ for 7d q4w) + magrolimab i.v. (30 mg/kg with ramp up on d1, 4,8,11,15, followed by q1w for 5 weeks and subsequently q2w administration q4w) vs. azacitidine s.c./i.v. + ventoclax p.o. OR “7 + 3” standard induction	ENHANCE‐2 NCT04778397	TP53^mut^ AML	III	258	Terminated due to futility. Primary outcomes of OS have not yet been reported.	N.a.	N.a.
Azacitidine s.c./i.v. (75 mg/m^2^ for 7d q4w) + magrolimab i.v. (30 mg/kg with ramp up on d1, 4,8,11,15, followed by q1w for 5 weeks and subsequently q2w administration q4w) + venetoclax p.o.(400 mg with ramp up for 28d q4w) vs. azacitidine s.c./i.v. + ventoclax p.o. + placebo i.v.	ENHANCE‐3 NCT05079230	Intermediate−/higher‐risk MDS	III	378	Terminated due to futility. Primary outcomes of CR and OS have not yet been reported.	N.a.	N.a.
Azacitidine s.c./i.v. (75 mg/m^2^ for 7d q4w) + sabatolimab i.v. (800 mg on d8 q4w) vs. azacitidine s.c./i.v. + placebo i.v.	STIMULUS‐MDS2 NCT04266301	Intermediate−/higher‐risk MDS and CMML	III	530	Trial completed, final results pending. Preliminary results of the primary endpoint mOS indicate no additional benefit of sabatolimab + azacitidine in comparison to sabatolimab + azacitidine.	N.a.	N.a.
Azacitidine s.c./i.v. (75 mg/m^2^ for 7d q4w) + sabatolimab i.v. (400 mg on d8,22 q4w) vs. azacitidine s.c./i.v. + placebo i.v. OR decitabine s.c./i.v. (20 mg/m^2^ for 5d q4w) + sabatolimab i.v. (400 mg on d8,22 q4w) vs. decitabine i.v. + placebo i.v.	STMULUS‐MDS1 NCT03946670	Intermediate−/higher‐risk MDS	II	127	Sabatolimab + DNMTi failed to demonstrate superiority over sabatolimab + placebo for the primary end points of CR rates (22% vs. 18%) and PFS (17.8 vs. 19.2 months).	N.a.	Zeidan AM et al. Lancet Hematology 2024: Sabatolimab plus hypomethylating agents in previously untreated patients with higher‐risk myelodysplastic syndromes (STIMULUS‐MDS1): a randomized, double‐blind, placebo‐controlled, phase 2 trial
Azacitidine s.c. (75 mg/m^2^ for 7d q4w) + durvalumab i.v. (1500 mg on d1 q4w) vs. azacitidine s.c.	FUSION‐AML‐001 NCT02775903	De novo AML (≥20% blasts) or secondary AML (due to MDS)	II	84	Augmented ORR of azacitidine + durvalumab compared to single agent azacitidine treatment (61.9% vs. 47.6%). No effect on mOS (11.6 vs. 16.7 months).	N.a.	Zeidan AM et al. Blood advances 2022: A randomized phase 2 trial of azacitidine with or without durvalumab as first‐line therapy for older patients with AML
Decitabine i.v. (20 mg/m^2^ for 10d q4w (cycle 1)) + midostaurin p.o. (50 mg bid d11‐28 q4w) vs. decitabine i.v.	HOVON155 2018–000047‐31	Higher‐risk MDS and AML	II	140	Decitabine and midostaurin failed to reach superiority over decitabine monotherapy in CR/CRi (24% vs. 34%), mOS (4.8 vs. 7.4 months) and 1‐year OS (31% vs. 37%)	N.a.	Huls G et al. Annals of Hematology 2024: Midostaurin added to 10‐day decitabine, for patients unfit for intensive chemotherapy with AML and higher risk MDS, irrespective of FLT3 mutational status, does not improve outcome
Azacitidine i.v. induction prephase (75 mg/m^2^ d‐5‐to d‐1) + cytarabine i.v. (100 mg/m^2^ d1‐7) and daunorubicin (60 mg/m^2^ d3‐5) q3w.	AML‐AZA SAL trial NCT00915252	AML	II	214	Study was terminated by the sponsor after predefined recruitment of 99% due to cardiac adverse events and drug shortage. The addition of azacitidine to standard “7 + 3” induction followed by HiDAC provided no additional benefit in EFS (6 months both) nor mOS (15 vs. 21 months) while increasing toxicities.	N.a.	C Müller‐Tidow et al. Leukemia 2016: Azacitidine in combination with intensive induction chemotherapy in older patients with acute myeloid leukemia: the AML‐AZA trial of the study alliance leukemia
G‐CSF s.c. (5‐10 μg/kg/d d‐17 to −10) + decitabine i.v. (20 mg/m^2^ d‐14 to d‐10) + busulfan i.v. (3.2 mg/kg/d d‐7 to d‐4) + cyclophosphamide i.v. (60 mg/kg/d d‐3 to d‐2) followed by allo‐HCT (d0) vs. busulfan i.v. + cyclophosphamide i.v. followed by allo‐HCT (d0)	NCT02744742	MDS (RAEB 1/2) and MDS related secondary AML undergoing allo‐HCT	III	202	Significantly lower 2‐year cumulative risk of relapse within the four‐drug allo‐HCT chemotherapy regimen compared to standard busulfan and cyclophosphamide (10.9% vs. 24.8%). The 100d post allo‐HCT non relapse mortality was comparable between the two cohorts, with similar incidence rates for infections (34% vs. 32%), GI‐toxicity (28% vs. 29%) and death (11% vs. 13%).	N.a.	L Xuan et al. Lancet Hematology 2023: The effect of granulocyte‐colony stimulating factor, decitabine, and busulfan–cyclophosphamide versus busulfan–cyclophosphamide conditioning on relapse in patients with myelodysplastic syndrome or secondary acute myeloid leukaemia evolving from myelodysplastic syndrome undergoing allogeneic hematopoietic stem‐cell transplantation: an open‐label, multicenter, randomized, phase 3 trial
Azacitidine s.c. (75 mg/m^2^ for 7d q4w) + durvalumab i.v. (1500 mg on d1 q4w) vs. azacitidine s.c.	FUSION‐AML‐001 NCT02775903	Intermediate−/very high‐risk MDS (<20% blasts)	II	84	No difference in ORR (61.9% vs. 47.6%) nor mOS (11.6 vs. 16.7 months) between the azacitidine + durvalumab cohort compared to single agent azacitidine. Combination therapy was able to induce PD‐L1 expression on bone marrow granulocytes and monocytes but not blasts.	N.a.	Zeidan AM et al. Blood Advances 2022: A randomized phase 2 trial of azacitidine with or without durvalumab as first‐line therapy for higher‐risk myelodysplastic syndromes

Abbreviations: “7 + 3”, cytarabine and anthracycline (daunorubicin/idarubicin/mitoxantrone) based standard induction therapy; allo‐HCT, allogeneic hematopoietic stem cell transplantation; AML, acute myeloid leukemia; ATRA, all‐trans‐retinoic acid; (c)CR, (composite) complete remission; CMML, chronic myelomonocytic leukemia; DNMTi, DNA methyltransferase inhibitors; DoR, duration of response; EFS, event‐free survival; EORTC QLQ‐30, European Organisation for Research and Treatment of Cancer quality of life questionnaire 30; EQ‐5D‐5L, European Quality of Life 5‐Dimensions 5‐Level questionnaire; FLT3^mut^, FMS‐related tyrosine kinase 3 ^(mutation)^; GI, gastrointestinal; HiDAC, high‐dose cytarabine; i.v., intravenous; IDH1^(mut)^, isocitrate dehydrogenase 1 ^(mutation)^; (m)OS, (median) overall survival; MDS, myelodysplastic syndrome; N.a., not applicable; ORR, overall response rate; p.o., per os; PD‐L1, programmed cell death 1 ligand 1; PROMIS Fatigue SF7a, Patient‐Reported Outcomes Measurement Information System Fatigue SF7a questionnaire; QOL‐ONE, Quality of Life—ONE questionnaire; s.c., subcutaneous; TP53^mut^, tumor protein p53 ^(mutation)^.

**TABLE 3:** Summary of regulatory approved DNMTi‐based single and combination therapies in hematologic malignancies.

Drug name	Mechanism	Method of application	Agency + year of approval	Approved indication
Azacitidine	DNMTi	s.c. or i.v.	FDA (2004) EMA (2008)	*FDA*: RA‐, RARS‐, RAEB and RAEB‐T MDS and CMML *EMA*: intermediate‐2 and high‐risk MDS (IPSS‐R), AML >20% blasts and CMML
Decitabine	DNMTi	i.v.	FDA (2006) EMA (2012)	*FDA*: de novo or secondary MDS of all FAB subtypes and intermediate‐1, intermediate‐2 and high‐risk MDS *EMA*: de novo or secondary AML not eligible for IC
CC‐486 (oral azacitidine)	DNMTi	p.o.	FDA (2020) EMA (2021)	*FDA and EMA*: AML in CR_(i)_ following IC and not eligible to complete intensive curation therapy
Cedazuridine/decitabine	CDAi/DNMTi	p.o.	FDA (2020) EMA (2023)	*FDA*: de novo or secondary MDS of RA‐, RARS‐, RAEB‐ FAB subtype or intermediate‐1, intermediate‐2, higher‐risk MDS and CMML *EMA*: newly diagnosed AML ineligible for IC
Azacitidine or decitabine + venetoclax	DNMTi + BCL‐2i	p.o. (venetoclax)	FDA (2020) EMA (2021)	*FDA*: newly diagnosed AML ≥75y years and ineligible for IC *EMA*: newly diagnosed AML ineligible for IC
Azacitidine + ivosidenib	DNMTi + IDH1i	p.o. (ivosidenib)	FDA (2018) EMA (2023)	*FDA*: IDH1^mut^ newly diagnosed AML ≥75y years or with comorbidities that preclude IC, IDH1^mut^ r/r AML, IDH1^mut^ r/r MDS *EMA*: newly diagnosed AML with IDH1^mut^ (R132) ineligible to receive IC

Abbreviations: AML: acute myeloid leukemia, BCL‐2i: B‐cell lymphoma 2 inhibitor, CDAi: cytidine deaminase inhibitor, CMML: chronic myelomonocytic leukemia, CR_(i)_: complete remission_(incomplete)_, DNMTi: DNA methyltransferase inhibitors, EMA: European Medicines Agency, FAB: French‐American‐British (classification for AML and MDS), FDA: U.S. Food and Drug Administration, IC: intensive chemotherapy, IDH1^(mut)^: isocitrate dehydrogenase 1 ^(mutation)^, IPSS‐R: Revised International Prognostic Scoring System (MDS scoring system), i.v.: intravenous, MDS: myelodysplastic syndrome, r/r: relapsed or refractory, RA: refractory anemia, RARS: refractory anemia with ringed sideroblast, RAEB: refractory anemia with excess blasts, RAEB‐T: refractory anemia with excess blasts in transformation, s.c.: subcutaneous.

In 2020, the combination treatment of azacitidine, decitabine, or low‐dose cytarabine in combination with venetoclax received full FDA approval for the treatment of newly diagnosed AML in adults who are 75 years or older, or in those with comorbidities that preclude IC based on the results of the VIALE‐A trial.^94^ This pivotal study, which involved 431 patients older than 75 years or ineligible for IC, demonstrated significant improvements in response rates and OS with the dual treatment compared to azacitidine or decitabine alone. After a median follow‐up of 20.5 months, the combination therapy resulted in an increased OS of 14.7 months (95% CI 11.9–18.7) compared to the OS of 9.6 months (95% CI 7.4–12.7) in the control regimen (HR 0.66, 95% CI 0.52–0.85, *p* < 0.001).^94^ The study also reported a higher rate of composite CR in the dual treatment arm (66.4%, 95% CI 60.6–71.9) compared to the control arm (28.3%, 95% CI 21.1–36.3, p < 0.001). Notably, the combination treatment demonstrated superiority in a subset analysis of patients with *IDH1/2, NPM1*, and *FLT3* mutations. Encouraging results have also been reported from a phase I/II study of azacitidine plus venetoclax in patients with high‐risk MDS or CMML.^95^ The confirmatory randomized phase III VERONA trial (NCT04401748) of venetoclax with azacitidine has been closed for accrual and is reported to be negative, though the publication is still pending (AbbVie press release 16th June 2025, NCT04401748).

#### 1.4.2 DNMTi in combination with retinoic acid

In contrast, the combination of DNMTi with many other targeted agents has yet, with very few exceptions, to show improved outcomes in randomized trials. DNMTi have also been studied in combination with agents that stimulate cell maturation. A randomized phase II study (DECIDER) of valproate or retinoic acid in combination with decitabine in elderly unfit AML patients revealed that all‐trans retinoic acid (ATRA) plus decitabine induced significantly higher remission rates than decitabine alone (21.9% vs. 13.5%, OR 1.80; 95% CI 0.86–3.79, *p* = 0.06) as well as significantly improved OS (8.2 vs. 5.1 months, HR 0.65, 95% CI 0.44–0.89, *p* = 0.006).^96^ Valproic acid had no effect in this trial. Based on this study, a randomized, placebo‐controlled, phase III trial (DECIDER‐2) investigating the effects of ATRA in combination with decitabine and venetoclax is ongoing (EUDRACT 2020‐005495‐36; DRKS 00023646, P001516).

Similar results have also been reported by a Chinese group investigating 223 MDS‐EB1/2 patients randomized 1:1 to decitabine and ATRA versus decitabine only, indicating significantly higher ORR (78% vs. 51%, OR 3.4, 95% CI 1.90–6.09, *p* < 0.001), higher CR rates (23% vs. 12%, OR 2.05, 95% CI 1.02–4.25, *p* = 0.042) and PFS (14.9 vs. 10.5 months, HR 0.70, 95% CI 0.51–0.97, *p* = 0.032). The SELECT‐MDS‐1 trial (NCT04797780), evaluating tamibarotene, an oral selective retinoic acid receptor α agonist, in combination with DNMTi was closed for futility.

#### 1.4.3 DNMTi in combination with ascorbic acid

In addition to clinical trials on vitamin A derivatives, investigating vitamin C, a cofactor of the epigenetic regulator *TET2*, has also sparked interest recently.^97^, ^98^ A phase II trial investigating a 12‐month oral 1000 mg/day vitamin C supplementation in lower‐risk MDS and MDS/MPN overlap patient cohorts has shown early positive results in OS, however, more extensive research will be needed to draw final conclusions.^99^

#### 1.4.4 Other DNMTi‐based dual combination studies

Combining several epigenetic drugs, thereby potentially eliciting a synergistic effect by the reactivation of silenced genes, has also sparked significant interest. Candidates include the histone deacetylase inhibitors (HDACi) entinostat and pracinostat, and the *IDH1* inhibitor ivosidenib.^100^ In the phase III PRUMULA trial, 406 AML patients were assigned 1:1 to receive azacitidine in combination with either pracinostat or placebo. Unfortunately, the trial failed to demonstrate superiority in both OS (both 9.95 months) and CCR (36% vs. 31.5%).^101^ In MDS, a phase II study reported similar outcomes with no significant differences in OS (16 vs. 19 months) and PFS (11 vs. 9 months).^102^ Likewise, the combination of azacitidine with another oral HDACi, entinostat, did not demonstrate improvement in hematological normalization (32% vs. 27%) nor ORR (46% vs. 44%) in MDS/AML patients when compared to single‐agent treatment only.^103^ Fortunately, the combination of other epigenetic drugs has been more successful. Ivosidenib, an oral *IDH1* inhibitor, was shown to augment 12‐month EFS (37% vs. 12%; HR 0.33 95% CI 0.16–0.69, *p* = 0.002) and improve OS (24.0 vs. 7.9 months; HR 0.44, 95% CI 0.27–0.73, *p* = 0.001) in a randomized, placebo controlled phase III clinical trial investigating newly diagnosed *IDH1* ^mut^ AML patients.^104^ Unsurprisingly, the most common AEs ≥3 grade were hematological and included febrile neutropenia and bleeding.

Beyond these dual epigenetic drug combinations, combination of DNMTi with several other, already well‐established single agents has also been exploited. In the open‐label, multicenter phase III LACEWING trial, 123 *FLT3* ^mut^ AML patients were randomized 2:1 to the *FLT3* inhibitor gilteritinib in combination with azacitidine or simply azacitidine.^105^ At an interim analysis, OS (9.82 vs. 8.87 months) and EFS (0.03 months in both) did not display a significant improvement, thereby leading to the termination of this trial (by set study criteria defined previously). Likewise, the first generation *FLT3* inhibitor midostaurin failed to demonstrate superiority over single agent decitabine treatment in inducing CR/CRi and 1‐year OS in a cohort of 140 higher‐risk MDS/AML patients.^106^ Ibrutinib, an oral Bruton's tyrosine kinase (BTK) inhibitor, has been investigated in a mixed MDS/AML patient cohort of 165 patients in combination with the DNMTi decitabine.^107^ No significant differences in OS (10.0 vs. 11.5 months) nor ORR (41% vs. 50%) could be reported. Additionally, bortezomib and lenalidomide, two very well‐established drugs in multiple myeloma, have undergone investigation, potentially repurposing them in the dual combination with an DNMTi. In a phase II trial of 165 AML patients, the addition of bortezomib to decitabine, however, did not improve OS (8.9 vs. 9.3 months) nor ORR (38% vs. 39%).^108^ Likewise, a combined phase II/III trial of the Northern American Study group investigated, in MDS and CMML, the effects of azacitidine with or without lenalidomide and vorinostat. ORR (38 vs. 49% vs. 27%) of the azacitidine only vs. azacitidine and lenalidomide vs. azacitidine and vorinostat cohort were similar.^109^

In addition to these well‐known therapeutics, novel drugs with new targets and mechanisms are quickly entering the stage, with some of them being investigated in combination with DNMTi in AML/AMS. Pevonedistat (TAK924, MLN4924) is a first‐in‐class, small molecule inhibitor of NEDD8‐activating enzyme that selectively prevents activation of Cullin‐RING ligases (CRLs) and thereby diminishes proteasomal degradation of CRL substrates. NAE inhibition demonstrated preclinical efficacy in multiple cancer models, including myeloid malignancies.^110^ Pevonedistat plus azacitidine was compared to azacitidine in higher‐risk MDS or low‐blast percentage AML.^111^ However, the phase III PANTHER trial failed to demonstrate improvements in EFS, or median OS, with only a subgroup analysis suggesting potential benefit in high‐risk MDS patients receiving more than three (23.8 vs. 20.6 months, *p* = 0.021) cycles or more than six cycles (27.1 vs. 22.5 months, *p* = 0.008).^112^ The most heavily investigated and targeted agent for patients with *TP53*‐mutant MDS and AML has been APR‐246 (eprenetapopt), a p53 reactivator, in combination with azacitidine, but also in triplets with venetoclax.^113^, ^114^ Despite positive results in phase II trials, a phase III trial did not confirm superior response or improved survival (NCT03745716).

### 1.5 DNMTi based‐combination with immune checkpoint inhibition

The dual combination of an antibody‐based backbone in conjunction with DNMTi has also been investigated intensely in recent years. Magrolimab, a first‐in‐class investigational monoclonal antibody against CD47 and macrophage checkpoint inhibitor is designed to interfere with recognition of CD47 by the SIRPα receptor on macrophages, thus blocking the “don't eat me” signal used by cancer cells. Despite positive results, the ENHANCE‐3 trial comparing magrolimab and azacitidine to azacitidine and placebo in untreated patients with MDS was terminated because of futility and excessive deaths in February 2024 (Gilead press release, NCT05079230). Bexmarilimab (FP‐1305) is a novel humanized anti‐CLEVER‐1 IgG4‐antibody capable of inducing a phenotypic M2 to M1 immune switch of tumor‐associated macrophages. It is studied in an ongoing phase I/II study in combination with azacitidine with or without venetoclax (NCT05428969) in MDS and AML.

Checkpoint inhibition in combination with DNMTi is the focus of several trials ongoing in MDS and AML. While early data appeared promising, confirmatory studies thus far have been largely disappointing. Sabatolimab, an experimental immunotherapy with immuno‐myeloid activity that targets TIM‐3 on immune cells and leukemic blasts, is being evaluated for the treatment of myeloid malignancies as part of the STIMULUS clinical trial program, in combination with azacitidine. However, the randomized phase III trial (NCT04266301) failed to reach superiority in OS in intermediate‐, high‐ or very high‐risk MDS not eligible for IC or allo‐HCT. Although data from this trial have not yet been published, preliminary results from the STIMULUS‐MDS2 trial appear to be consistent with the negative findings of the STIMULUS‐MDS1 trial, which evaluated the combination of azacitidine with sabatolimab in previously untreated higher‐risk MDS patients.^115^

Two randomized phase II trials investigating azacitidine with or without durvalumab (anti‐PD‐L1) as first‐line therapy for older, frail patients with higher‐risk MDS (<20% blasts) or AML (≥20% blasts) demonstrated that the addition of durvalumab did not improve ORR (61.9% vs. 47.6% for MDS, 31.3% vs. 35.4% for AML) or OS (11.6 vs. 16.7 months for MDS, 13.0 vs. 14.4 months for AML) compared to single agent azacitidine treatment.^116^, ^117^ Nivolumab (anti‐PD‐1) with or without ipilimumab (anti‐CTLA‐4) is being studied in a non‐randomized phase II study in patients with MDS. The study is closed for accrual, however results are pending (NCT02530463). The currently ongoing LEAP trial (NCT03092674) is a randomized controlled phase II/III clinical trial investigating various treatments for older patients with newly diagnosed AML or MDS. Per design of this study, azacitidine is either administered as single agent (arm A), combined with either nivolumab (arm B) or midostaurin (arm C), whereas in arm D, 5‐day decitabine is followed by cytarabine. The study is expected to finish recruiting by late 2025.

Pembrolizumab (anti‐PD‐1) is being studied in a phase Ib trial in combination with decitabine with/without venetoclax, for r/r MDS or AML. Interim results of six patients with AML demonstrated that four patients responded to this dual treatment (NCT03969446), final results are awaited. Another phase II study of azacitidine with pembrolizumab in patients with intermediate‐1 or higher‐risk MDS, including patients who were deemed DNMTi refractory, displayed only moderate activity.^118^ A study of atezolizumab (anti‐PD‐L1) in conjunction with azacitidine was terminated early by the sponsor due to high early death rate and lack of efficacy.^119^

### 1.6 DNMTi in lymphoid malignancies

While azacitidine and decitabine are firmly established as a therapeutic backbone in MDS and AML patients who cannot tolerate IC, their use in lymphoid malignancies, specifically non‐Hodgkin lymphoma (NHL) and chronic lymphocytic leukemia (CLL), is exploratory and thus off‐label. Clinical trials indicate potential benefits, particularly in combination therapies, but these applications require further validation through larger, randomized studies. Several studies have explored the combination of azacitidine (and, less frequently, decitabine) with lenalidomide in CLL and NHL, both sequentially and concurrently.^120^, ^121^ In a phase II study of decitabine in r/r aggressive NHL, the hypomethylating agent was well‐tolerated and exhibited modest clinical activity, with an ORR of 40% versus 33% for the standard of care, though this difference was not statistically significant.^120^ In addition, others have tried to boost the efficacy of standard lymphoma therapy (R‐CHOP) by combining this treatment with a DNMTi. A phase I study of 59 patients in intermediate/high‐grade diffuse large B‐cell lymphoma showed potential clinical benefit by combining oral CC‐486 with standard R‐CHOP. ORR was 94.1% (88.1% in CR) with a 1‐year PFS of 84.1%.^122^ However, larger follow‐up trials are needed to further validate these results.

### 1.7 DNMTi in solid tumors

DNMTi have been investigated for the treatment of solid tumors since the 1970s, with the most notable agents being decitabine and azacitidine. These compounds have shown some potential, particularly when used in combination with other therapeutic agents. However, clinical outcomes have been variable, and ongoing research is critical to better define their role and optimize their therapeutic efficacy.

#### 1.7.1 Lung cancer

Preliminary pilot studies in the 1990s by Momparler et al. suggested that decitabine may demonstrate clinical efficacy in treating metastatic non‐small cell lung cancer, NSCLC.^123^ However, a phase I/early‐phase II clinical trial combining cisplatin and decitabine in patients with metastatic and inoperable NSCLC did not demonstrate significant clinical benefit.^124^ Conversely, a phase II trial using decitabine as a priming agent before chemotherapy in advanced NSCLC showed that the combination of decitabine and chemotherapy demonstrated some clinical benefit, with a disease control rate of about 60%.^125^ Despite this, the OS benefit was modest.

#### 1.7.2 Ovarian cancer

In recurrent, platinum‐resistant ovarian cancer, several small trials have been conducted.^126^, ^127^, ^128^, ^129^ A phase II trial of decitabine followed by carboplatin demonstrated that low‐dose decitabine could sensitize tumors to carboplatin, leading to an ORR of 35%.^127^ The combination was well tolerated, exhibiting manageable toxicity. In another phase II clinical trial, which enrolled 100 patients, epigenetic priming was assessed by comparing guadecitabine in combination with carboplatin to standard TC chemotherapy in recurrent, platinum‐resistant ovarian cancer.^129^ While this study showed improved PFS at 6 months, it did not significantly impact median PFS.

#### 1.7.3 Breast cancer

In breast cancer, DNMTi have been studied preclinically since 2006, often in combination with other therapeutic modalities such as HDACi and tamoxifen, summarized in a review by Schröder et al.^130^ Preclinical results have been promising, with one study suggesting re‐expression of estrogen receptor after application of a dual combination of HDACi and DNMTi in triple‐negative breast cancer (TNBC) cell lines,^131^ while another study demonstrated reversibility of tamoxifen resistance in cell lines upon azacitidine treatment.^132^ There are also promising preclinical data for guadecitabine plus HDACi in a TNBC model.^133^ However, early clinical trials conducted in the 1970s failed to report an additional benefit of azacitidine in breast cancer.^134^, ^135^ Furthermore, despite preclinical evidence of synergy between DNMTi and HDACi, a clinical study published in 2017 found only one responder in a series of 40 patients with either TNBC or hormone refractory breast cancer treated with the combination of azacitidine and entinostat, an HDACi.^136^ The combination was well tolerated, with biomarker studies indicating re‐expression of silenced genes; however, clinical efficacy was limited. A recent study reported on pre‐neoadjuvant treatment in advanced breast cancer with pembrolizumab and decitabine.^137^ The study found higher levels of stromal tumor‐infiltrating lymphocytes (sTILs) after administration of the two agents prior to standard neoadjuvant therapy. Higher sTILs in breast cancer are associated with an increased likelihood of pCR. A modest, yet statistically significant increase in sTILs could be demonstrated in a subset of 46 patients evaluated.

#### 1.7.4 Colorectal cancer

In previously treated metastatic colorectal cancer, guadecitabine was evaluated in combination with irinotecan in a phase I and randomized phase II clinical trial.^138^, ^139^ The phase II trial failed to demonstrate a benefit of the combination over standard of care in terms of response rates and PFS.^139^

#### 1.7.5 Melanoma

The combination of oral azacitidine with pembrolizumab in advanced melanoma demonstrated promising anti‐tumor activity.^140^ In checkpoint inhibitor‐naive patients, the initial response rate was approximately 55%. However, an immune checkpoint‐pretreated cohort did not respond to this treatment, supporting the hypothesis that azacitidine can modulate the tumor microenvironment to enhance immune response in immune checkpoint treatment‐naïve patients.

## 2 CONCLUSIONS AND OUTLOOK

The clinical development of DNMTi for the treatment of patients with myeloid neoplasias has been moving steadily forward over the last four decades, with continuous improvements regarding low‐dose development, scheduling, modes of administration, extension of treatment duration and, importantly, the combination with cooperating agents, as well as the use of “bridging” to subsequent allo‐HCT.

During the early phases of the development of low‐cytotoxic doses and schedules of azacitidine and decitabine, clinical studies were primarily focused on MDS patients who were elderly and not medically fit to tolerate more intensive treatment. Administration of either drug in these early trials was complex, particularly for decitabine, where patients were hospitalized to receive six daily 2‐h infusions over a total of 72 h (hence with infusions administered also at midnight and at 2 AM). In several of these trials, the total number of treatment cycles to be given was limited, thereby not allowing for prolonged maintenance treatment to stabilize and thus prolong remissions in responding patients. In addition, DNMTi regimens historically followed traditional pulse‐cycled models used for other anti‐metabolic treatments; however, their subsequent recognition of low‐dose molecular targeted DNMT depletion guided the development of currently used low‐dose regimens.^100^ Regarding their particular mechanism of action and distinct clinical activity, it is now well accepted that these agents induce in vivo gene de‐methylation and de‐repression of silenced genes; importantly, also the—at first surprising and counterintuitive—highly reproducible observation of recurrent responses in patients with complex‐monosomal karyotypes and *TP53* mutations is now accepted, and DNMTi‐based drug combinations are aimed at improving the outcome of these patients despite the formidable challenges of this genotype.

At present, DNMTi have been FDA‐approved as monotherapy for high‐risk MDS, with doses and schedules that are feasible on an outpatient basis, with an FDA approval also for the oral formulation of decitabine in conjunction with cedazuridine. In AML, the combination of a DNMTi with venetoclax in patients ineligible for standard intensive induction chemotherapy was approved by both FDA and EMA. For AML and MDS patients ineligible for the curative approach of allo‐HCT, continued treatment with a DNMTi, alone or in combination with venetoclax, may stabilize complete remissions over years. A metronomic, very low‐dose treatment of decitabine (alone or with venetoclax) administered weekly has also resulted in very promising, remarkably well‐tolerated results.^141^

As shown in the EORTC Intergroup study AML21, in patients aged >60 years and fit for standard induction chemotherapy, a de‐escalation approach investigating monotherapy with an extended 10‐day decitabine schedule resulted in similar overall survival, similar transplant rate (overall >50%), and survival from transplant as standard IC, albeit with a lower rate of toxicities, better quality of life, reduced numbers of hospitalizations, use of antibiotics, and antimycotics. This trial thus provides a blueprint for the ongoing (NCT04801797, NCT05904106, NCT05177731) and future studies randomizing fit AML patients, also those below 60 years of age, for either IC or a hypomethylating agent in combination with venetoclax. Attempts to challenge the current standard of care IC with a de‐escalation approach in the frontline setting for younger, medically fit AML patients are currently ongoing. Lu et al. provided compelling evidence that a venetoclax‐decitabine dual treatment is non‐inferior to IC in regard to CCR rates with similar MRD‐negativity and OS, while simultaneously reducing treatment‐related AE such as infections and thrombocytopenia.^142^ It is to be hoped that the de‐escalation strategy of this “less is more” approach will supersede traditional IC in different AML subgroups.

Relapse treatment after allografting is frequently approached by DLI, that is, cellular immunotherapy, with hypomethylating agents, achieving disease control in the majority of patients, and in a subgroup even has curative potential. DNMTi are also developed for the treatment of MPN, MDS/MPN overlap syndromes, and juvenile myelomonocytic leukemia (JMML): azacitidine is FDA approved as first‐line treatment in JMML (a myeloid cancer of childhood with a very poor prognosis unless treated with DNMTi followed by allografting).

The goal to improve the survival of elderly, medically non‐fit patients has also driven the implementation of QoL assessments before and during treatment. Quantification of patients' time not spent in the hospital and with good quality of life has also prompted QoL assessments in younger AML/MDS patients, raising awareness for the important objective of the reduction of “time toxicity” in hematology‐oncology (and medicine in general). In that regard, it is intriguing that de‐escalating treatment intensity, without sacrificing efficacy, that is, the starting point of the development of low‐dose DNMTi treatment for elderly non‐fit patients 40 years ago, may now convey benefit also for younger patients with myeloid malignancies.^142^ It is to be hoped that this trend will also benefit patients with lymphoid neoplasias and solid tumors in the future.

## AUTHOR CONTRIBUTIONS

**Valentin Wenger:** Writing – review and editing; writing – original draft; conceptualization; investigation. **Guillermo Garcia‐Manero:** Writing – review and editing. **Robert Zeiser:** Writing – review and editing; writing – original draft; funding acquisition. **Michael Lübbert:** Writing – review and editing; writing – original draft; funding acquisition; project administration; supervision; conceptualization; investigation.

## CONFLICT OF INTEREST STATEMENT

Valentin Wenger has received honorarium from AbbVie. Robert Zeiser has received honoraria from Novartis, Incyte, VectorBio, Medae, Sanofi, and Therakos. Michael Lübbert is on the advisory boards of AbbVie, Astex, Janssen, and Otsuka, is receiving research support from Janssen, and is provided with study drug by Cheplapharm. Guillermo Garcia‐Manero reports no conflicts of interest.
